# Novel gene discovery for hearing loss and other routes to increased diagnostic rates

**DOI:** 10.1007/s00439-021-02374-0

**Published:** 2021-10-01

**Authors:** Hannie Kremer

**Affiliations:** 1 Hearing and Genes, Department of Otorhinolaryngology, Radboud University Medical Center, Nijmegen, The Netherlands; 2 Department of Human Genetics, Radboud University Medical Center, Internal Postal Code 855, P.O. Box 9101, 6500 HB Nijmegen, The Netherlands; 3 Donders Institute for Brain, Cognition and Behaviour, Radboud University Medical Center, Nijmegen, The Netherlands

## Abstract

Despite decades of research, there is much to be learned about the genetic landscape of sensorineural hearing loss. Novel genes for hearing loss remain to be identified while ‘secrets’ of the known genes need to be uncovered. These ‘secrets’ include regulatory mechanisms of gene activity and novel aspects of gene structure. To obtain a more complete picture of the genetics of hearing loss, the available experimental and bioinformatic tools need to be fully exploited. This is also true for data resources such as ENCODE. For the inner ear, however, such data resources and analytical tools need to be developed or extended. Collaborative studies provide opportunities to achieve this and to optimally use those tools and resources that are already available. This will accelerate the discoveries that are necessary for improving molecular genetic diagnostics and genetic counselling and for the development of therapeutic strategies.

## Introduction

It is clear from the missing diagnoses in molecular genetic testing as well as from deafness loci for which the defective genes are still elusive that our knowledge of the genetic landscape of (nonsyndromic) sensorineural hearing loss is far from complete (Hereditary Hearing Loss Homepage (HHL Homepage, https://hereditaryhearingloss.org/); Vona B et al. 2019). The main reasons for missing defects underlying hearing loss with a presumed genetic cause include limitations of the employed technologies and of tools for variant interpretation, as well as our incomplete knowledge of the structure and regulatory mechanisms of genes already known to be associated with hearing loss. A further important reason for missing diagnoses in routine molecular genetic testing is that the catalogue of genes known to be associated with hearing loss is incomplete. This is confirmed by a steady pace of gene discoveries for hearing loss (HHL Homepage; Vona et al. 2019). However, besides that deafness loci can harbour elusive genetic defects, it should also be considered that a locus resulted from innocent methodological mapping errors, genetic heterogeneity in families, or phenocopies among affected family members.

## Identification of novel genes for hearing loss

It is an open question how many genes for hearing loss await to be identified. For mouse, it is estimated that about 1000 genes are critical for hearing (Ingham et al. 2019) which suggests a comparable exceptional genetic heterogeneity for hearing loss in humans, as well. How should we effectively complete the gene catalogue for hearing loss? The predominant strategy for the genes that have been identified since early 2019, was whole exome or genome sequencing (WES, WGS) in multiple members of families with hearing loss segregating in an autosomal dominant or recessive pattern. This was preceded or complemented by linkage analysis or homozygosity mapping in several of the studies and functional effects of variants were demonstrated in cell models. To confirm that the genes were indeed associated with hearing loss, either animal models were studied (mouse, zebrafish, fruitfly) or cohorts of index cases were screened to identify additional pathogenic variants. A second important approach was screening of index cases for variants in candidate genes that were found to be critical for hearing in knock out mice from the International Mouse Phenotyping Consortium (IMPC; Dickinson et al. 2016). Such candidate genes have also been employed to prioritise variants identified in family studies. Ideally, both animal models and cohort studies are combined to confirm the association of a gene with a hearing loss phenotype. In addition, in vitro or ex vivo experiments, biochemical assays, and/or cell biological assays can provide evidence for pathogenicity of variants in novel genes for hearing loss.

Multiplex families and candidate genes are indeed excellent sources for the identification of novel genes for hearing loss (see article by Acharya, this issue). As there is a positive correlation between the number of investigated genes and the diagnostic yield (Vona et al. 2019), it is important to accelerate gene identification and to confirm associations of those genes for which the evidence is insufficient for reporting their variants in molecular genetic testing. Decreasing costs of genome analyses allow to increase the numbers of index cases and families that can be studied. Importantly, this creates excellent opportunities to make major steps forward in large collaborative studies as such studies reveal study cohort(s) and datasets with sufficient power for novel analysis strategies. Innovative meta-analysis tools can be applied that need to be either specifically designed or adapted for hearing loss. Meta-analyses have been successfully employed to identify genes for intellectual disability although the predominance of de novo mutations in this condition reduces the complexity of such an analysis (Kaplanis et al. 2020; Lelieveld et al. 2016). Large cohorts will also allow their stratification for specific analyses e.g. based on the presumed inheritance pattern of the condition and functional effect of causative variants (e.g. loss of function effect in recessively inherited hearing loss), or onset age and characteristics of the hearing loss to finetune meta-analysis strategies or enrich for specific genetic defects. A further benefit of joining forces is that this provides opportunities to implement cost-effective candidate gene analysis and pre-screening strategies of the known genes for hearing loss. This is relevant for those cases that were tested previously only for a subset of genes and for cases for which the possibilities for genetic testing are limited. Unsolved cases can subsequently enlarge patient cohorts for further studies. In a recently established consortium, called Solve-RD, both data and expertise are shared to diagnose patients with rare diseases (Zurek et al. 2021). Solve-RD could serve as a blueprint for a consortium on hearing loss. Funding to establish a consortium for hearing loss would be an excellent way to provide more research groups and/or diagnostic laboratories access to WES and WGS. The importance to collaborate and to apply WES or WGS also provides opportunities to urge significantly increased resources for research in countries and communities in which these are too limited.

## Scrutinising (known) genes for hearing loss

An important open question is whether identification of novel genes for hearing loss or scrutinising the known genes will have the largest impact on the diagnostic rates. Several types of genetic variation of these known genes are currently not (effectively) addressed and/or difficult to interpret. This is specifically the case for variants in the noncoding regions of genes and flanking regulatory regions including sequences that determine (tissue-specific) chromatin organisation. Tools to predict aberrant transcript splicing caused by (deep-)intronic variants are being used and predicted effects are often experimentally tested in mini-gene splice assays. Evaluation of variants in noncoding DNA for a potential deleterious effect on gene regulation is more challenging. Modelling of variants in conserved sequences in mouse can provide insights into a (potentially) pathogenic effect and mechanism (see article by Bowl, this issue). This is also illustrated for a small deletion in *HGF* intron 5 that was predicted to occur in the 3’UTR of an alternatively spliced transcript (Morell et al. 2020). Such extensive studies are only possible for a limited number of variants, and likely to be initiated if there are strong indications for deleteriousness. Our insight into the regulatory landscape of gene expression is limited, especially for the inner ear, which hampers variant interpretation. Emerging resources and tools such as the ENCODE (the Encyclopedia of DNA Elements) dataset (ENCODE; Project Consortium et al. 2020), and DESCARTES (the Developmental Single Cell Atlas of Gene Regulation; Cao et al. 2020; Domcke et al. 2020) will increasingly facilitate to address variants in regulatory regions, although the inner ear was not analysed yet in these projects. In other projects, studies of the inner ear are being performed and data on gene expression and chromatin accessibility are becoming available (Muthu et al. 2019; Yizhar-Barnea et al. 2018; gEAR portal [https://umgear.org/]). An experimental strategy to obtain clues to potentially pathogenic effects of noncoding variation on either transcript splicing or gene regulation is to combine WGS and RNA sequencing data of affected subjects. However, this is only meaningful for those genes that are expressed at a sufficient level in accessibly cell types such as fibroblasts and peripheral blood cells. In the latter cell source, this is true for about 30% of the known genes for hearing loss (the Genotype-Tissue Expression (GTEx) project and K. Neveling, personal communication). Analysis pipelines for this strategy have been developed and successfully applied *e.g.* for mitochondriopathy patients (Kremer et al. 2017). This approach can also shed light on potential effects of (missense or synonymous) variants of unknown significance (VUS) on transcript splicing or of structural variants such as inversions and translocations that are often missed in short read sequencing. Such structural variants can, however, be effectively identified by long read genome sequencing and Bionano optical mapping (Chan et al. 2018). Obviously, inner ear-specific effects on transcript splicing and gene expression will not be detected with the indicated approaches. Options to elucidate such effects are iPSC-derived inner ear cells that are being more broadly implemented (see article by Romano, this issue). The relevance of technologies in molecular genetic testing for hearing loss is reviewed in de Bruijn et al. (2021).

In the Deafness Variation Database, more than 79% of variants in genes implicated in hearing loss are classified as VUS, most of which are missense variants (Azaiez et al. 2018), despite the availability of a plethora of tools to predict potential deleterious effects. Obviously, developing strategies to evaluate the pathogenicity of these VUS may reveal a major increase of the diagnostic yield. Addressing enrichment of such variants in large (population-specific) cohorts of index cases as compared to controls can provide valuable insights but many variants are ultra-rare. Analysing family members for segregation patterns of such variants remains important but is often difficult. Studying potential enrichment of variants in specific gene regions might enhance variant interpretation. Also, bioinformatic tools such as MetaDome (Wiel et al. 2019) can be explored for their effectivity in variant interpretation in genes for hearing loss or for selection of missense variants to ultimately perform functional evaluation at the protein level. Such functional assays still need to be developed for most of the hearing loss associated genes which is not a trivial task (see article by Miyoshi, this issue). The same is true for the implementation of such assays in clinical practice.

Last but not least, variants in known genes for hearing loss with an allele frequency higher than expected based on disease incidence or prevalence need to be considered for a pathogenic effect with incomplete penetrance. This might be specifically relevant for genetic types of hearing loss with a variable, mainly adult onset and/or variable severity. The occurrence of reduced penetrance of variants as well as the potential genetic complexity of a Mendelian disorder is exemplified in clinically and allelically highly heterogeneous *ABCA4*-associated retinal degeneration (Cremers et al. 2020). The genetic complexity is illustrated by an extremely hypomorphic missense variant with an allele frequency of ~ 7% in Europe which is completely penetrant when present *in cis* with an additional variant. Also, the existence of (non-)genetic modifiers is indicated part of which might have a gender-specific effect on disease penetrance.

All discussed aspects for the evaluation of variants in genes already known to be associated with hearing loss are also relevant for the identification of novel genes for hearing loss. Therefore, the knowledge and expertise gained by analysing the full range of genetic variation of these known genes will facilitate the identification of novel genes that are critical for hearing in humans. The analysis of known genes as well as the identification of novel genes for hearing loss can be importantly accelerated and supported by initiatives such as building population-specific databases for hearing loss and the development of hearing loss-specific multi-omics baseline data.

## Non-Mendelian inheritance patterns in hearing loss

Large WES and/or WGS datasets of genetically unexplained index cases as well as families will enable us to address non-Mendelian inheritance patterns of hearing loss, more specifically digenic or oligogenic inheritance. So far, there is no strong evidence of digenic inheritance of hearing loss. However, non-Mendelian inheritance might well significantly contribute to hearing loss in isolated cases for which the diagnostic yield is significantly lower than for familial cases. This is also true for adult-onset hearing loss even if this is familial. The genes involved might already be associated with hearing loss but could also be novel. The recently developed platform ORVAL (the Oligogenic Resource for Variant AnaLysis) aims to identify candidate pathogenic variant combinations in gene pairs (Renaux et al. 2019) and it would be interesting to explore this for hearing loss.

In conclusion, identification of novel genes for hearing loss is an ongoing process that is importantly facilitated by the developments in DNA and RNA sequencing technologies and in tools for bioinformatic and experimental analysis of genetic variation, as well as by the emerging resources of *e.g.* regulatory elements. The same technologies and tools will also facilitate the identification and interpretation of variation in the known genes for hearing loss and non-Mendelian inheritance patterns. Important steps forward can be made in global collaborative frameworks designed to exchange expertise, enlarge patient cohorts and combine datasets to increase the power of analysis tools that are already available or to be developed. The ongoing efforts to characterise the genetic landscape of hearing loss in many different populations, as presented in several articles in this issue (*e.g.* Naz, this issue) are already paving the way for global collaborative efforts.
